# Towards Tailored Gut Microbiome-Based and Dietary Interventions for Promoting the Development and Maintenance of a Healthy Brain

**DOI:** 10.3389/fped.2021.705859

**Published:** 2021-07-01

**Authors:** Ana Larroya, Jorge Pantoja, Pilar Codoñer-Franch, María Carmen Cenit

**Affiliations:** ^1^Microbial Ecology, Nutrition & Health Research Unit, Institute of Agrochemistry and Food Technology, National Research Council (IATA-CSIC), Valencia, Spain; ^2^Department of Pediatrics, University Hospital De la Plana, Vila-Real, Castellón, Spain; ^3^Foundation for the Promotion of Health and Biomedical Research in the Valencian Region (FISABIO), Valencia, Spain; ^4^Department of Pediatrics, Dr. Peset University Hospital, Valencia, Spain; ^5^Department of Pediatrics, Obstetrics and Gynecology, University of Valencia, Valencia, Spain

**Keywords:** gut microbiota, gut bacterial microbiome, virome, diet, precision nutrition, neurodevelopment, psychiatry and mental health

## Abstract

Mental health is determined by a complex interplay between the Neurological Exposome and the Human Genome. Multiple genetic and non-genetic (exposome) factors interact early in life, modulating the risk of developing the most common complex neurodevelopmental disorders (NDDs), with potential long-term consequences on health. To date, the understating of the precise etiology underpinning these neurological alterations, and their clinical management pose a challenge. The crucial role played by diet and gut microbiota in brain development and functioning would indicate that modulating the gut-brain axis may help protect against the onset and progression of mental-health disorders. Some nutritional deficiencies and gut microbiota alterations have been linked to NDDs, suggesting their potential pathogenic implications. In addition, certain dietary interventions have emerged as promising alternatives or adjuvant strategies for improving the management of particular NDDs, at least in particular subsets of subjects. The gut microbiota can be a key to mediating the effects of other exposome factors such as diet on mental health, and ongoing research in Psychiatry and Neuropediatrics is developing Precision Nutrition Models to classify subjects according to a diet response prediction based on specific individual features, including microbiome signatures. Here, we review current scientific evidence for the impact of early life environmental factors, including diet, on gut microbiota and neuro-development, emphasizing the potential long-term consequences on health; and also summarize the state of the art regarding the mechanisms underlying diet and gut microbiota influence on the brain–gut axis. Furthermore, we describe the evidence supporting the key role played by gut microbiota, diet and nutrition in neurodevelopment, as well as the effectiveness of certain dietary and microbiome-based interventions aimed at preventing or treating NDDs. Finally, we emphasize the need for further research to gain greater insight into the complex interplay between diet, gut microbiome and brain development. Such knowledge would help towards achieving tailored integrative treatments, including personalized nutrition.

## Introduction

Neurodevelopmental disorders (NDDs) are linked to the disruption of coordinated events leading to neurodevelopmental processes, and constitute a cluster of disorders characterized by the inability to reach cognitive, emotional, and motor developmental milestones (1). Although NDDs have an early onset, they frequently persist into adulthood, potentially leading to significant long-term health consequences. Characteristically, NDDs constitute a serious health problem in our society, affecting roughly 3% of children worldwide. In addition, the prevalence of these conditions is rising, which poses a serious challenge.

Human genetics is a well-known key factor involved in the etiology of the most common NDDs; however, both environmental and gene-environment interactions appear to strongly influence their clinical manifestation, which would explain the lack of heritability of these disorders (1, 2). Despite many years of research, studies have failed to fully identify all determinants and mediators leading to the onset and progression of these multifactorial conditions and, thus, their precise etiologies remain uncertain. High comorbidity among NDDs is frequently observed, which in turn worsens their prognosis (3). In this regard, the identification of the shared pathogenic mechanisms among the different NDDs will ultimately help to lead to effective treatment to combat this high comorbidity as well as to monitor their progress and to anticipate future complications.

The diagnosis of these conditions, based mainly on clinical symptoms, and their management is highly challenging. Pharmaceutical treatment, the first-line therapy of choice for clinical management, is often associated with several side effects, and other concerns about long-term effects and efficacy exist (4). Therefore, new alternatives are required for the diagnosis, prevention and treatment of these disorders. In this respect, dietary intervention ranks very highly among such strategies and has the added benefit of being acceptable by most people as more “benign” and potentially free of side effects.

The bidirectional nature of the gut–brain axis is well-recognized, with gut microbiota emerging as a key player controlling this two-way communication system (5). Importantly, the gut microbiota establishes its symbiotic relationship with the host early in life and the initial perturbations of this connection can impact neurodevelopment, potentially leading to adverse mental health consequences later in life (6). In-depth knowledge of this critical early microbial–neural window of opportunity will open new insights for novel microbiota modulation-based therapeutic interventions in early life, targeting neurodevelopmental impairments and brain disorders (7). In fact, recent evidence suggests that dysregulation of the gut-brain axis may be a crucial factor contributing to many mental health related disorders. In this regard, animal models such as germ-free (GF) mice, entirely devoid of microbiota, as well as animals treated with antibiotics or with specific bacterial species have shed light on how the gut microbiota and its genome (gut microbiome) are involved in regulating brain development and function and, consequently, cognition and behavior (8, 9).

Specifically, studies using GF mice have established that the total absence of microbiota impairs social behavior (10), as well as other types of behavior such as anxiety and stress response (11–13). Furthermore, certain behaviors linked to the lack of gut microbiota correlate with neurochemical changes in different brain regions, with specific alterations in neurotransmitter pathways (14). Additionally, fecal microbiota transplantation has demonstrated its ability to transfer certain behavioral traits, suggesting a causal link and thus pointing to alterations in the microbiota as a trigger of particular phenotypes (15). Moreover, a growing number of human cross-sectional studies have investigated the microbiota composition in individuals with certain neurological disorders or impairment vs. healthy age-matched individuals, and identified gut microbiota alterations associated with neurological alterations. However, these studies only provide a snapshot in time, which is a strong limitation to establishing causality (9) and, thus, there is a clear need for well-designed human prospective studies to identify the specific human gut microbial members or gut microbiota profiles able to trigger NDDs or to modify their prognosis.

Diet has been recognized as one of the main factors shaping the gut microbiota and consequently its modulation is considered one of the easiest ways to develop and maintain a healthy gut microbiome. In fact, gut microbiome modulation through dietary interventions has been widely suggested as a robust and attainable strategy to prevent disorders, including mental conditions (16–18), and to improve human health (19, 20). In this regard, modulation of gut microbiota functionalities has been proposed as a key mediator of the dietary effects on mental health (21). However, diet is also known to be involved in brain development and functioning through both microbiota-dependent and independent mechanisms (22). Thus, scientific evidence indicates that diet and gut microbiota can interact or act synergistically to provide resilience against disorders, or conversely work together in the progression from health to disease.

In the context of NDDs, some dietary interventions, including dietary advice and specific diets or certain dietary components and supplements, may influence the onset and manifestation of neurodevelopmental impairments in childhood neurological disorders, at least in specific cohorts (23–27). However, a high inter-individual variation in the clinical response to different dietary interventions has been well-documented, and patient subgroups with the same diagnosed condition respond differently to certain dietary interventions (26). Accordingly, within the framework of Nutritional Psychiatry, research has focused on “precision nutrition,” which is currently working on the design of tailored microbiome-based and dietary intervention strategies to promote the development and maintenance of a healthy brain (26–29). It is important to note that potential new tailored dietary-based intervention strategies could pave the way to preventing the development of NDDs in subjects with risk indicators and constitute also new alternatives for improving the clinical management of subjects diagnosed with NDD.

In this context, we should highlight that the relationship between diet and mental health is complex and bidirectional, and studies report less healthy dietary choices among patients with NDDs (30, 31). In this regard, subjects suffering NDDs might also find it more difficult to adhere to potentially beneficial dietary interventions. Therefore, there is an urgent need to decipher the mechanisms by which dietary intervention exerts beneficial effects, with a view to designing safer and easy-to-implement targeted intervention strategies for patients with mental disorders.

Here, we review the current scientific evidence for the impact of early life environmental factors on gut microbiota and neurodevelopment, and highlight the long-term consequences of early nutrition on physical and mental health. We also summarize the state of the art regarding the mechanisms by which gut microbiota influences the brain–gut axis. In addition, we describe the evidence supporting the key role played by gut microbiota, diet and nutrition in neurodevelopment, as well as the effectiveness of certain dietary and microbiome-based interventions aimed at preventing and treating NDDs. Finally, we highlight the need for further research to advance towards achieving essential tailored integrative treatments, including personalized nutrition.

## Impact of Early Life Enviromental Factors on Gut Micriobiota and Neurodevelopment: Key Role of Diet

One of the main promises of the Human Genome Project (HGP) was to increase our understanding of the mechanisms underlying the etiology of complex diseases, thereby furthering their prevention and treatment. However, after HGP completion, human genetics was found to explain merely 20–30% of the etiology of these traits, with the rest owing to environmental factors and their interactions with the human genome (32).

The exposome can be defined as the measure of all types of exposure an individual is subjected to in a lifetime and include insults from the external environment, which interact with the internal environment (biological factors such as metabolism, gut microbiota, inflammation, and oxidative stress) impacting on health (33, 34). The host is especially vulnerable to the effects of environmental risk factors during organ development in the prenatal period, infancy, childhood and adolescence. Consequently, the early life Exposome-Genome interplay leads to crucial long-term consequences on mental and overall health (35). Additionally, it is well-established there are periods where gut microbiota is more unstable and, therefore, at greater risk of suffering alterations due to exposure to environmental stressors. These periods can also be considered as windows of opportunity to positively influence mental health through gut microbiota modulation (7).

Remarkably, the critical gut microbiota developmental period occurs in parallel with brain development (2) and, although it may experience changes later in life despite greater resilience, gut microbiota colonization of the infant is more dynamic. This process begins prenatally, and the symbiotic link between the host and the microbiota is established early in life during the perinatal period and continues through infancy, childhood and early adolescence (7).

**Figure 1:**
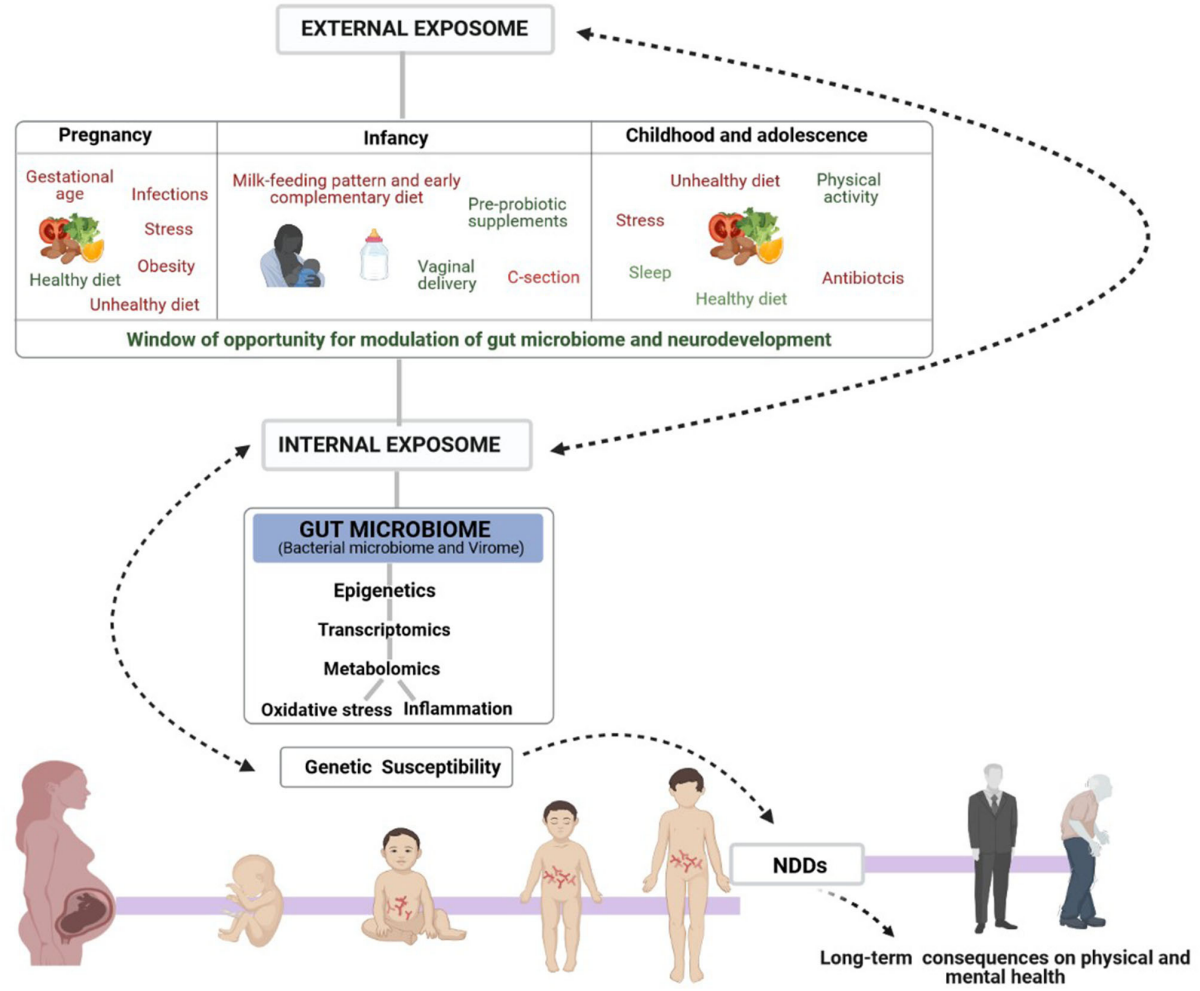
Schematic representation of the complex Exposome-Genome interplay in human neurological development. The figure also shows the early window of opportunity for gut-microbiome and neurodevelopmental modulation to influence mental health, mainly through dietary interventions. Early non-genetic risk and protective factors of the external exposome (prenatal, infancy, childhood and adolescence lifestyle factors) interact with the internal exposome (biological factors such as gut microbiota, metabolism, inflammation, and oxidative stress) controlling the onset of the most common NDDs in genetically susceptible individuals and consequent physical and mental health in adult life.

Well-documented factors associated with the risk of NDD onset and contributors to the individual's microbiota are those related to maternal health (stress, infections, gestational age, obesity and exposure to medication, including antibiotics and other drugs) (36) and many others related to infant and childhood health (37–41) such as mode of delivery, early type of feeding, medication, stress and infections (Figure 1). In fact, maternal health, diet and nutrition during pregnancy can modulate the offspring microbiome and neurodevelopment as well as brain characteristics of the offspring, both positively and negatively, by regulating neurotransmitter pathways, synaptic transmission and signal-transduction pathways (42). Evidence from animal models has demonstrated that maternal high-fat-diet (MHFD)-induced obesity alters gut microbiota composition in the offspring and impacts behavioral programming, thereby triggering impairments in social/emotional behavior and cognitive development (43). Recent studies in mice have shown that some probiotics reverse the effects of stress caused by maternal separation (42) while timely dietary interventions can rescue maternal obesity-induced behavioral deficits and neuroinflammation in offspring (44, 45).

By contrast, several studies have associated maternal adherence to a Mediterranean diet with favorable offspring behaviors. Important to note it is that many of the Exposome factors playing a role in the etiology of NDDs, including diet, are known to be closely related with the risk of developing disorders or conditions through epigenetic mechanisms (46, 47). Mode of delivery has also been reported as one of the main regulators of the early-life gut microbiota composition, and a recent study in mice demonstrated that alterations in gut microbial community induced by Cesarean-section (C-section) are associated with anxiety and social and cognitive impairment in the offspring (48). In this regard, the authors also reported that supplementation from birth with a *Bifidobacterium* breve strain, or with a dietary prebiotic mixture that promotes bifidobacterial growth, reverses selective behavioral alterations in C-section mice. These results strongly suggest that adjunctive microbiota-targeted therapies may be developed to help avert the long-term negative behavioral consequences associated with C-section. In consistency with this, very recently in a human prospective study it has been reported the impact of breastfeeding on the modulation of the longitudinal impact of delivery mode on the gut microbiota composition, with an observed increase in the relative abundance of *Bacteroides fragilis* and *Lactobacillus* in Cesarean-delivered infants with longer duration of breastfeeding (49). This finding strongly supports the fact that duration of breastfeeding might plays a critical role in restoring a health-promoting microbiome.

Other environmental exposures such as antibiotics administration during pregnancy, in the first months of life, childhood or adolescence may also strongly impact gut microbiota colonization and determine neurocognitive impairments later in life (39–41, 50). A recent study on the effects of 3-weeks microbiota depletion in antibiotic-treated mice reported that transient microbiota depletion during adolescence, a particularly vulnerable time for gut microbiota composition and when the brain is highly responsive to certain environmental factors, has long-lasting effects on microbiota composition and increases anxiety-like behavior; however, this was not the case in adulthood (51).

Nonetheless, the precise positive and negative consequences of gut microbiota manipulation, mainly through dietary interventions in adolescent mice, have yet to be fully understood, and require more in-depth study. In this respect, human dietary interventions have already emerged as promising alternatives or adjuvant therapies for the prevention and clinical management of NDDs, at least for specific subsets of patients (23–27). However, further human trials should be conducted to investigate the precise role of dietary modification in rescuing behavioral and cognition alterations induced in early life, and to understand whether gut microbiota modulation mediates these effects. Currently, the potential beneficial effects of early-life dietary supplementation with omega-3 polyunsaturated fatty acids (PUFAs) on neurocognitive development are being extensively explored. To date, research has shown the importance of omega-3 PUFA supplementation during the prenatal and early-life period as a potential protective factor for the onset of neurological impairments (52–54).

It is important to highlight that the full understanding of the early interaction between gut microbiota, environment and host through well-designed prospective human studies at early stages in life would open new insights for microbiome-based therapeutic interventions. Such strategies could prevent the development of NDDs in individuals at risk, or improve the treatment of NDD diagnosed subjects.

## Gut Microbiota Mechanisms Modulating Gut-Brain Axis

**Figure 2:**
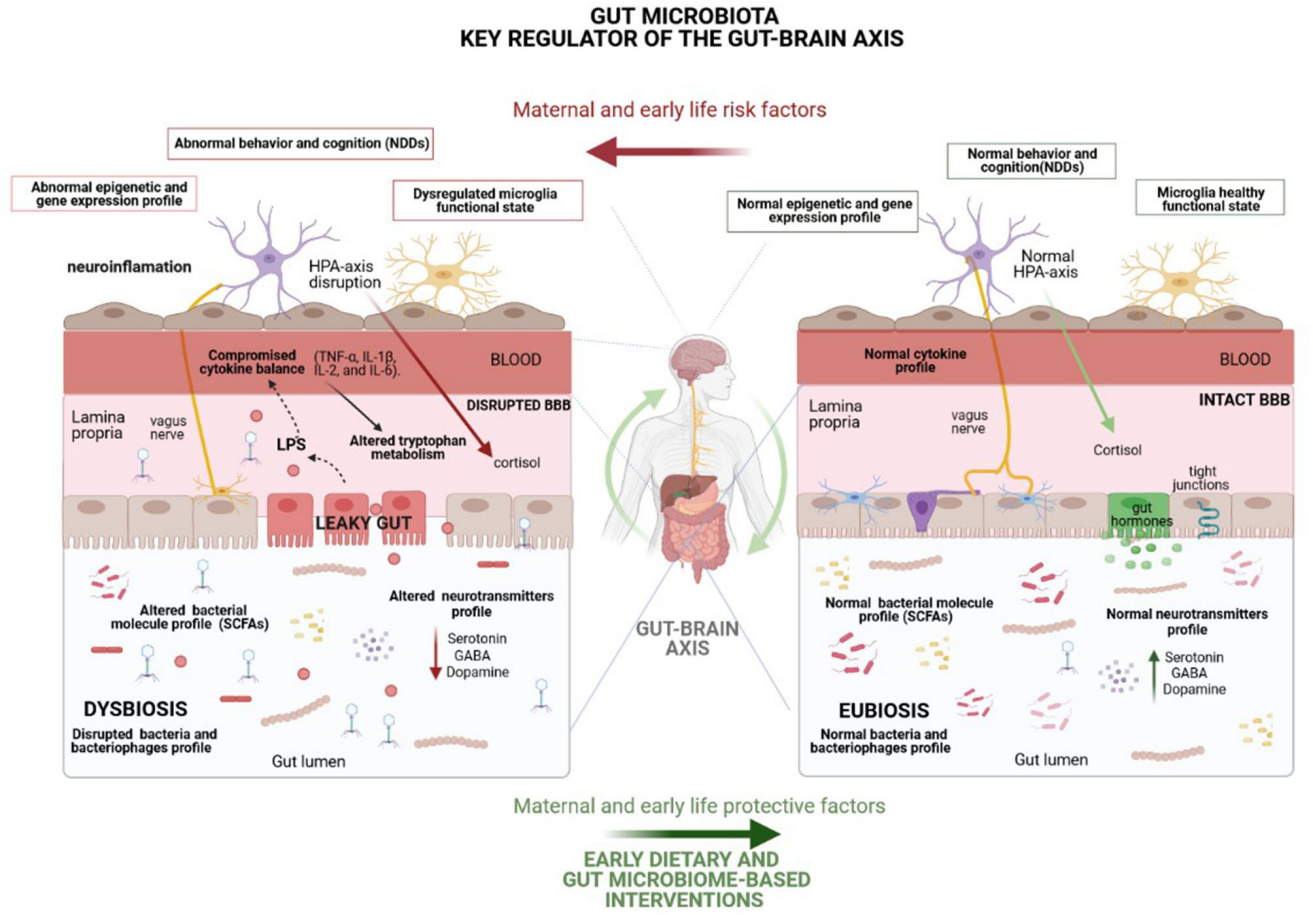
Representation of the underlying mechanisms, including immune (cytokine balance and microglia function), metabolic (short-chain fatty acids), endocrine (cortisol) and neural (vagus and enteric nervous system-ENS) pathways, by which the gut microbiota modulates the gut–brain axis and, consequently, brain function and behavior. Many environmental factors may disturb gut microbiota homeostasis, leading to a microbial imbalance (dysbiosis) which enhances the risk of NDDs mainly through gut permeability disruption, as well as by the disturbance of the host stress hormones and cytokine profile and the alteration of neurotransmitter metabolism and levels of other crucial neuroactive molecules. Highlighted potential of healthy dietary interventions to curb the harmful effects of life stressors on gut microbiota composition and mental health. HPA, Hypothalamic-pituitary-adrenal; BBB, Blood brain barrier.

Although further understanding of how the microbiota regulates gut-brain communication is required in order to establish the rationale behind microbiota-based interventions, numerous mechanisms by which gut microbiota regulates the gut–brain axis and subsequently modulates brain development and function have been described, including immune, metabolic, endocrine and neural pathways (9) (Figure 2). Remarkably, gut microbiota can modulate eating behavior by, for example, influencing reward and satiety pathways (55), which may, in turn, potentially change microbiota composition and function (56).

The gut microbiota can substantially impact on host metabolomics profile. In this context, certain gut microbiota members produce or modulate the levels of different neuroactive molecules, such as essential neurotransmitters and other specific neuromodulators (5). Neurotransmitters in the intestinal lumen can induce epithelial cells to release molecules, including hormones and cytokines, potentially modulating neural signaling within the enteric nervous system and, consequently, controlling brain function, cognition and behavior. Additionally, the brain can influence gut microbiota composition and function through the release of hormones or neurotransmitters, which act on gut physiology and microbiota environment, resulting in preferential growth of certain microbial communities (5). Some animal studies have also shown that different bacterial strains mediate their effects on brain function and behavior via the vagus nerve. Regarding neurotransmitters, tryptophan is an essential amino acid precursor of the neurotransmitter serotonin and also the precursor of metabolites related to the kynurenine pathway, whose metabolism is controlled by gut microbiota (57). Furthermore, the downstream metabolites of the kynurenine pathway have neuroactive properties and can also modulate neurotransmission (5).

Furthermore, some studies in germ-free (GF) mice have revealed that gut microbiota modulate the development of the HPA axis, impacting stress response. In this respect, some studies have proven the effectiveness of microbiota-based interventions in increasing stress resilience in healthy subjects (58). Other signals to the brain that originate from the gut microbiome are likely mediated through a variety of bacterial neuroactive metabolites, such as short-chain fatty acids (SCFAs) butyrate, acetate, and propionate, which bind free fatty acid receptors in the brain. Specifically, in rats, the administration of high doses of propionate, a common preservative found in foods, induces neuroinflammation and behavioral alterations associated with NDDs (59).

Research has also revealed that the gut microbiota largely impacts host gene expression by modulating epigenetic modifications, such as methylation, histone acetylation or non-coding RNAs (ncRNAs), which are involved in neurogenesis, neuronal plasticity, learning and memory (60). Thus, modulation of the microbiota might enable us to shape our epigenome. Indeed, methylation levels of genes involved in inflammatory responses have been linked to gut microbiota profiles. Several studies have defined a link between the levels of specific microRNAs and microbiota (61), while others have also reported that SCFAs can inhibit histone deacetylases (HDACs), activating the gene expression of previously deacetylated genes that impact on crucial physiological processes (60). In this regard, butyrate has been demonstrated to exert beneficial behavioral effects, through an epigenetic mechanism related to histone deacetylase inhibition (62).

The gut microbiota is well-known to play a central role in modulating oxidative stress as well as immune system development and function (63, 64). The involvement of the relationship between dietary constituents, gut microbiota and gut immunity has been emphasized in the overall homeostasis of inflammation and oxidative stress, pathways with important implications on the central nervous system and involved in the mechanisms of action and therapeutic effects of omega-3 PUFAs and other dietary supplements on mental health (65). In addition, dysregulation of microglial functions, such as synaptic pruning, are involved in disorders associated with cognitive impairment and gut microbiota plays a crucial role in the development and function of this specialized macrophage cell population in the central nervous system (66). In fact, the administration of SCFAs has been reported to rescue the microglial function previously compromised in GF animals (67).

Through the effect of SCFAs, the microbiota has also been shown to modulate the levels of brain-derived neurotrophic factor (BDNF), a neurotrophin with important effects on brain development and plasticity, which is dysregulated in patients with NDDs (68). Therefore, further studies are warranted to explore whether gut microbiota could be modulated to positively impact neurotrophin levels in the context of neurodevelopment (69). Finally, it is also important to note the role of gut microbiota in modulating gut permeability, as well as the permeability of the blood-brain barrier by regulating the expression of tight-junction proteins (70).

## The Potential Role of the Underexplored Virome in Neurodevelopment

Nowadays, it is clear that the human gut is inhabited not only by bacteria but also by less explored gut prokaryotic and eukaryotic viruses. With the growth of next-generation sequencing technologies, our knowledge about the role of gut bacterial microbiome in health and disease has rapidly expanded (71). By contrast, the role of the genome of gut viruses (gut viral microbiome or virome) on health still remains underexplored due to the important challenges facing gut virome research. Notwithstanding, in recent years these microorganisms have gained their own “omics,” becoming known as (meta)viromics (72).

In studying the role of viruses, researchers have traditionally focused on eukaryotic viruses due to their well-known impact on human health. A few years ago they began to realize the relevance of gut bacteriophages for gut microbiome dynamics, and to pay attention to the previously ignored relationship between bacteriophages and human diseases. In fact, cumulative evidence has started to suggest that gut bacteriophages known as phages, which are prokaryotic viruses that dominate over other viruses in the gut ecosystem and infect gut bacteria, can also strongly impact human health, either beneficially or detrimentally (72). In fact, despite the challenges facing research into the role of the virome in health, compelling evidence is emerging for bacteriophage involvement in several diseases, such as Inflammatory bowel disease (IBD) (73), Type 1 diabetes (T1D) (74), Type 2 diabetes (T2D) (75) and Clostridium difficile infection (CDI) (76), among others. Through predation, bacteriophages can influence the abundance of specific bacterial taxa, with indirect effects on the rest of the microbial community. Moreover, in certain cases, bacteriophages can be responsible for driving bacterial evolution to adopt more or less virulent phenotypes, by inserting their genomes into the bacterial chromosome (77). The human gut virome develops rapidly after birth and during early development and, like the bacteriome, it is tremendously dynamic (78) with gut virome inter-individual variability largely due to environmental rather than genetic factors (79). Specifically, recent research reports an expansion of the eukaryotic virome and the bacterial microbiome from birth to 2 years of age, during healthy development, closely accompanied by a decrease and shift in bacteriophage composition (78). Additionally, in recent years it is becoming evident that alterations in the virome early in life, similarly to early alterations in the bacterial microbiome, could set the stage for the infant's future health and disease risk. In this regard, although there is still a knowledge gap in mother–infant virome transmission, various studies have indicated that mode of delivery, breastfeeding and early nutrition are among the main environmental factors involved in shaping the infant virome (78).

Furthermore, ongoing studies are indicating the high potential of bacteriophages to modulate bacteria linked to the intestinal mucosa and related to diseases (80, 81). For instance, IBD patients seem to display a significant expansion of bacteriophages from the order Caudovirales (75), while Cornault et al. found that prophages of Faecalibacterium prausnitzii, a bacterium repeatedly found depleted in IBD patients, are more abundant in the fecal samples of IBD patients compared to healthy controls (82). Since IBD patients generally have less *F. prausnitzii* in their gut microbiota than healthy controls, the higher abundance of some of phages infecting major human gut commensal bacteria, such as *F. prausnitzii*, may indicate the potential role of these phages in IBD pathophysiology (75). Additionally, recent scientific evidence indicates that the virome profile at the time of transplant can influence treatment success of FMT. In this regard, bacteriophage transfer during FMT in Clostridium difficile infection has been associated with treatment outcome (83). These findings pave the way for further studies investigating whether bacterial microbiome modulation through bacteriophages may improve the effectiveness of therapeutic strategies, such as dietary interventions. In relation to mental health, extensive evidence has supported the fact that exposure to viruses can alter cognition and behavior (84, 85). Furthermore, several common human herpesvirus and some bacteriophages, including the lactobacillus phage phi-adh, are more predominant in individuals with schizophrenia compared to controls, and their abundance has also been associated with different clinical patterns and medications (84). In particular, the high abundance of a chlorovirus, specifically Acanthocystis turfaea chlorella virus-1 (ATCV-1), has been associated with decreased cognitive functioning in both mice and healthy subjects without an underlying psychiatric disorder (85). ATCV-1 exposure in mice was also found to result in an altered expression of genes involved in pathways related to synaptic plasticity, learning and memory, in the hippocampus (85).

Thus, it is important to highlight the need for further investigation into the role of the virome in shaping the gut ecosystem during early life, as well as the influence of external factors on the long-term evolution of bacteriome–virome interactions, and the involvement of these interactions in neurodevelopment and mental health. In addition, we have yet to elucidate the precise mechanisms through which viruses could influence human health, including brain functioning. Greater knowledge about the role of the virome in brain development and function may promote future approaches toward more accurate manipulation of the human gut microbiota through the modulation of viruses to improve mental health.

## Gut Microbiota, Behavior and Cognition

The gut microbiota is known to play a critical role in essential brain processes such as myelination, neurogenesis and microglial activation involved in behavior, mood and cognition (14). Several studies using GF mice have demonstrated that gut microbiota is key to social behavior (10) and other types of behavior such as anxiety (86) and stress response (87). Moreover, the ability to transfer behavioral traits by FMT supports the fact that some microbiota changes could rather be a driver rather than a consequence of the behavioral alteration (15). Additionally, recent studies using GF animals have also shown that the microbiome can control neurogenesis (88), a key process in learning and memory, as well as function of the amygdala (89), a brain area for social and fear-related behaviors, and prefrontal cortical myelination (90). On the other hand, administration of certain probiotics to healthy rats and mice can modulate behavior, accomplishing beneficial effects on stress-related behaviors by reducing anxiety-like and depressive-type behaviors (91). The gut microbiota assembly and dynamic progression in early life is shown to be accompanied by dramatic changes in the brain and the emergence of a number of cognitive functions (7). However, the effect of diet and gut microbiota on the development and progression of cognitive functions is still largely unknown (92). To date many studies in animal models have indicated the short-term and long-term effects of diet and eating habits on cognitive functions. For instance, a high-fat diet, which changes gut microbiota composition leading to gut dysbiosis, has been widely associated with cognitive decline, while experimental manipulation of gut microbiota in rodents has been shown to impact on cognition (93). Proper HPA-axis functioning has been demonstrated essential for cognitive processes, such as memory and learning, and specific microbiota components beneficially modulate stress-induced alteration of the HPA axis (94).

However, similar evidence in humans is scarce. An observational study investigating 77 children at 18–27 months of age showed some associations between gut microbiome composition and child temperament characteristics, specifically reporting a positive correlation of extraversion and gut microbiota diversity in both boys and girls, as well as an association between fear and a high abundance of *Rikenellaceae* in girls (95). Regarding gut microbiome and cognition, a cross-sectional study reported a positive correlation between gut microbial composition and adult cognition (96). A specific association has been described between the 1-year-old gut microbiome and cognitive outcomes at one and 2 years of age (97). Particularly, a prospective study predicted better cognitive development in infants with higher alpha gut microbiota diversity, as well as with higher abundance of *Bacteroides* putative beneficial microorganisms, which have been linked to vaginal delivery (97). However, studies have not yet addressed when exactly this relationship between gut microbiota and cognition emerged.

## Gut Microbiota, Diet and ADHD

Attention deficit hyperactivity disorder (ADHD) is currently the most prevalent neurodevelopmental complaint. It is characterized by inappropriate levels of hyperactivity, impulsivity and/or attention difficulties (98). Current ADHD standard treatment is based on a multimodal approach combining psychotherapy and pharmacotherapy. However, in clinical trials 20–35% of ADHD subjects showed an inadequate response to the treatment (99). Moreover, pharmacotherapy is frequently associated with adverse side effects and its long-term efficacy is still questionable (100). Therefore, safer and alternative treatments for ADHD are clearly needed. In this regard, several environmental factors, including diet, associated with the risk of developing ADHD, are linked to shifts in gut microbiota composition, which suggests a link between gut microbiota and ADHD etiology (101). In fact, some studies have found gut microbiota alterations linked to ADHD. However, findings provided by these studies have been largely inconsistent and the specific gut microbiota members or profiles involved in ADHD onset and prognosis have yet to be elucidated (102). To date, there is no evidence on whether gut microbiota-directed interventions can help in ADHD management, although preclinical evidence on gut microbiota-ADHD causality has recently been reported for fecal microbiota transplant in mice (103). Regarding the potential effects of probiotics on neurobehavioral outcomes in children and adolescents, a recent systematic review reported that prenatal and early infant probiotic supplementation with *Lactobacillus rhamnosus GG (LGG)* is associated with a lowered abundance of gut *Bifidobacteria* in infancy, and a decreased risk of developing ADHD (104).

Research into the effect of food on ADHD started (105) 40 years ago when pediatric allergist Benjamin Feingold hypothesized that both artificial food additives and foods rich in salicylates would contribute to ADHD development and manifestation. Later, the Feingold studies were followed by other elimination diet studies, investigating the effects of either artificial food color (AFC) elimination or of a diet eliminating numerous foods and additives, called the few-foods diet (FFD), and by studies investigating the effects of supplements, vitamins, minerals, and PUFAs on ADHD (106). Some of these studies showed the protective effect of increased intake of specific nutrients such as iron, zinc, iodine and PUFAs, while others confirmed the adverse effect of excessive ingestion of food coloring agents, preservatives and sugar. In addition, different double-blind placebo-controlled studies, investigating the effect of a FFD on ADHD, provided consistent evidence for the fact that ADHD might be triggered by particular foods, at least in a subgroup of subjects (26). As a result of these studies, the potential benefit of certain dietary interventions was proposed for the prevention and treatment of ADHD.

More recently, meta-analyses have evaluated the efficacy of dietary interventions on children with ADHD. They showed that the clinical effects of PUFA supplementation and food additive elimination were small to modest, while the effects of the FFD on ADHD were substantial, pointing to the existence of a food-induced subtype of ADHD (105). Indeed, the FFD approach is often applied in clinical practice, and a recent study showed its effectiveness, leading to a clinically relevant reduction of ADHD and Oppositional Defiant Disorder (ODD) symptoms (26). Additionally, recent meta-analyses reported that omega-3 PUFA supplementation seems to provide small benefits for ADHD symptoms (106), effects that seem to be largely modulated by different genetic and non-genetic factors. On the whole, these alternatives offer new opportunities for non-treated ADHD or for children with ADHD who do not respond to medication.

Unhealthy dietary patterns and ADHD show a positive correlation (31), suggesting that healthy dietary patterns might protect against or successfully treat ADHD, while an unhealthy diet might potentially increase ADHD risk. Consistent with this, two studies recently reported an association between ADHD and low adherence to the Mediterranean diet, suggesting that the Mediterranean diet might help prevent ADHD development (107, 108). The relationship between diet and mental health is bidirectional, which often implies unhealthy dietary choices among patients with mental disorders. Thus, the consumption of unhealthy foods might be a consequence of ADHD rather than a determinant of the disorder, since certain foods, especially those with high sugar content, are activators of the reward system and consequently lead to high intake (109). In fact, several studies have shown a higher prevalence of eating disorders (110, 111) and obesity (112) in individuals with ADHD, which often implies the greater difficulty of these subjects to adhere to potentially beneficial dietary interventions. So far, there is not irrefutable evidence about diet causality on ADHD and, in this regard, future studies are needed. Longitudinal and interventional randomized controlled trials, instead of cross-sectional designs, should be implemented to investigate the potential beneficial effect of a healthy diet on ADHD symptomatology, and to generally investigate the causal role of diet in ADHD onset and manifestation.

Besides the reported ADHD-diet association, there is also scientific evidence for an association between other lifestyle factors and ADHD. According to research, children with ADHD are almost twice as likely to demonstrate less healthy behavior, which leads to the previously described association between ADHD and obesity, even after adjustment for other multiple parameters such as age, sex, medication and comorbidities (113). Given this close association between ADHD and obesity, clinicians should initiate nutrition counseling with families of children with ADHD shortly after diagnosis to prevent obesity and its comorbidities. In this regard, generally improved lifestyle choices may provide more substantial benefits to children with ADHD than dietary modifications alone and, thus, future research is required to assess the effects of a combined healthy lifestyle-diet intervention on ADHD (113).

Observational studies have also demonstrated some associations between specific micronutrient levels in subjects with ADHD and the presence or severity of symptoms; however, these studies do not allow conclusions to be drawn on causal relationships. In addition, as demonstrated in omega-3 PUFAs supplementation, the therapeutic benefits of supplementation may depend on baseline nutrient status and thus may likely be narrowed to individuals with specific micronutrient deficiencies (114). Furthermore, the Ketogenic diet has recently been suggested as a dietary intervention with potentially therapeutic effects for ADHD, warranting greater investigation into its role in prevention and treatment of the disorder.

## Gut Microbiota, Diet and Social Behavior: Focus on Autism Spectrum Disorders

Autism spectrum disorders (ASDs) are neurodevelopmental disorders characterized by the presence of stereotypical behavior, exhibiting communication and social interaction deficits. Although the precise ASD etiology remains unknown, both genetic and environmental factors are recognized to play a key role in its pathogenesis. Many environmental factors linked to the risk of developing ASDs have been associated with gut microbiota shifts (dysbiosis) as dysbiosis-related gastrointestinal alterations are often associated with ASDs. Furthermore, a strong positive correlation has been demonstrated between ASD severity and the severity of the comorbid gastrointestinal dysfunction linked to gut dysbiosis (115). Regarding investigation into the role of gut microbiota in social interaction and communication, it is important to highlight that studies have demonstrated that GF-mice display impairment in social behavior and increased repetitive traits. These alterations are normalized following bacterial colonization. This fact strongly suggests that a healthy composition of gut microbiota is essential for normal social behavior and that autism-like symptoms would be favored when normal gut bacterial species are absent (10). In addition, fecal microbiota transplantation from human donors with ASD or from typically developing (TD) control subjects into GF mice also revealed that the colonization with ASD gut microbiota is sufficient to induce autistic behavior in mice (116). In particular, the mice with gut microbes from ASD donors showed lower levels of specific compounds produced by gut bacteria, such as the amino acids 5-aminovaleric acid (5AV) and taurine, which affect brain function by increasing the activity of the brain's γ-aminobutyric acid (GABA) receptors. Interestingly, the transfer of these two missing metabolites (5AV and taurine) to mice with autism-like symptoms was also reported to improve core deficits in social interaction and repetitive behavior. Cryan *et al*. also reported that mice with an autism-like condition showed lower levels of *Bifidobacteria* and *Blautia* gut bacteria, and their guts produced fewer compounds, such as tryptophan and bile acid, needed to produce serotonin (117). It is important to note that we require a greater understanding of the mechanisms underlying the social deficits, which may include modulation of immune cell cytokine release, changes in vagal nerve activity and neuroendocrine function. Such knowledge could potentially lead to the emergence of novel and more effective therapies to counteract symptoms in the social domain.

The administration of a single bacterial strain can modulate the social behavior of animals beneficially. Either *Bacteroides fragilis* (70) or *Lactobacillus reuteri* (118) could reverse many of the behavioral and gastrointestinal alterations reported in ASD. Interestingly, a study using the maternal immune activation (MIA) mouse model (animal model known to display features of ASD as well as gastrointestinal barrier alterations and microbiota changes) demonstrated that the oral treatment of offspring with the human commensal *Bacteroides fragilis* was able to correct gut permeability, restore microbial composition, and ameliorate alterations in communicative, stereotypic, anxiety-like and sensorimotor behaviors (70). The same study also revealed that MIA offspring displayed an altered serum metabolomic profile, and that *B. fragilis* supplementation was able to positively modulate levels of several metabolites (70). These findings strongly indicate the strong potential of probiotic therapy for GI and specific behavioral symptoms linked to neurodevelopmental disorders, such as ASD.

Many studies in humans have shown gut microbiota alterations in ASD subjects. Despite the lack of consistency in some of the gut microbiome changes reported across studies, various cross-sectional studies comparing the gut microbiome of neurotypical children vs. children with ASDs have reported increased levels of Clostridiales in the latter. In addition, Luna *et al*. showed increased levels of *Clostridiales* (*Lachnospiraceae* and *Ruminococcaceae*) in ASD children with gastrointestinal pain (119). Furthermore, children with autism have also consistently shown lower levels of *Veillonellaceae, Coprococcus*, and *Prevotella* gut bacteria than those without the condition. Additionally, cumulative causal evidence from preclinical studies has revealed that gut microbiota and SCFAs seem to play an important role in gastrointestinal disorders and ASD. In this regard, Liu *et al*. recently observed a decreased abundance of key butyrate-producing taxa (*Ruminococcaceae, Eubacterium, Lachnospiraceae, and Erysipelotrichaceae*) in autistic children, together with low levels of SCFAs detected in patients' fecal samples (120), suggesting that butyrate-producing bacteria could be a promising strategy for ASD treatment. On the other hand, *Clostridium perfringens* strains isolated from the microbiota of children with ASDs were found to express the gene encoding b2 toxin, cpb2, to a greater extent than the same strain isolated from the microbiota of neurotypical children (121). This toxin is associated with various gastrointestinal diseases, which may help to explain the comorbid gastrointestinal symptoms frequently observed in ASD individuals. Furthermore, Clostridia bacterial pathogens have been reported to generate propionic acid in the gut, a compound known to disrupt the production of neurotransmitters and to causes autism-like symptoms in rats (122). Of note, whereas animal studies have reported promising results for the direct connection between early life gut microbiota and neurobehavioral development related to ASD, data in human populations reporting causality are still scarce, with only two recent prospective studies supporting a potential causal association between infant gut microbiome and early childhood neurodevelopment (123, 124).

In this regard, a very recent study has explored this causal association by analyzing gut microbiota and measuring early childhood neurodevelopment with the Ages and Stages Questionnaire, third edition (ASQ-3), and suggests that the infant gut microbiome may be associated with subsequent development of communication, personal and social, and fine motor skills in 3-year-old children and with delays. In fact, the main finding was the potential association between *Clostridiales* and poorer communication, personal and social scores, and with increased likelihoods of delay in the development of these skills (124). Consistent with these results, another recent study aimed to explore the direct link between gut bacteria and the onset and manifestation of ASD. The authors also found potential associations between the early-childhood gut microbiome and social behaviors. By investigating the associations between the infant/toddler gut microbiome at 1, 2, and 3 years and ASD-related social behaviors at the age of 3 years, several taxa were found to be associated with the Social Responsiveness Scale 2 (SRS-2) performance, including many in the *Lachnospiraceae* family. Particularly, higher relative abundance of *Adlercreutzia equolifaciens* and *Ruminococcus torques* at 1 year were associated to poorer SRS-2 performance (123). One small-scale pilot study of microbiota-transfer therapy in patients with ASD also showed promising results (125). Children with ASD receiving microbiota transfer therapy were reported to have significantly reduced gastrointestinal disruptions, and significant improvements in ASD-related behavior, which persisted for at least 2 years after the end of treatment. The authors also noted a significantly reduced bacterial diversity and significantly increased abundance of *Bifidobacterium, prevotella*, and *desulfovibrio* after treatment.

Some studies investigating the effects of potential probiotics in subjects with ASD have reported promising results. Indeed, several studies suggest that probiotic supplementation in children with ASD might reduce intestinal inflammation and permeability, and restore the abnormal gut microbiota linked to ASD, leading to an improvement in GI and ASD symptoms. Particularly, a recent 4-weeks, randomized, double-blind, placebo-controlled study investigated the effects of *Lactobacillus Plantarum PS128* on ASD symptoms, a psychobiotic strain with beneficial effects on anxiety, and demonstrated that a 28-days intervention with the probiotic significantly ameliorated certain behavior, specifically opposition/defiance, in boys with ASD (126).

Regarding the potential use of probiotic supplementation for ASD prevention, in a recently published review (104) researchers reported only one study demonstrating that specific probiotic supplementation with *Lactobacillus rhamnosus GG* during pregnancy and during the first year of life was associated with a reduction in the risk of developing Asperger's syndrome (127). It is worth noting that the reduction in disease onset risk was coupled with a lowered abundance of gut *Bifidobacterium* in infancy and, although the mechanisms underlying the benefits of this probiotic supplementation remain unclear, a potential explanation lies in the modulation of gut microbiota composition through probiotic supplementation. Furthermore, the administration of the prebiotics galacto-oligosaccharides (GOS) and fructo-oligosaccharides (FOS) increases social interaction in chronically stressed mice, positively influencing the relative abundance of the key bacterial genera *Akkermansia, Bacteroides, and Desulfovibrio* (128). However, further human intervention studies are warranted to determine whether probiotic/prebiotic supplementation can be useful for pregnant mothers as preventive tool for ASD.

Regarding nutritional deficiencies linked to the onset of ASDs, several preclinical studies have reported that early life dietary deficiency of omega-3 fatty acids leads to deficits in social recognition (129). Conversely, omega-3 supplementation during childhood and adolescence has been demonstrated to improve social behavior in both animal and human studies (130). Particularly, a recent pilot trial found a significant improvement in ASDs symptoms after omega-3 supplementation in preterm children showing early signs of ASD (131). Additionally, a variety of other dietary deficiencies, including deficiencies of vitamins A, C, B6, B12, D, and folate (132), have also been suggested as potential triggers of ASDs onset. As a result, observations that these deficiencies may be linked to ASDs have led to several trials to study the effects of supplementation on treating ASD symptoms. Remarkably, multiple studies have also documented both reduced methylation and antioxidant biomarkers, such as S-adenosylmethionine and glutathione (GSH), in individuals with ASD. Specifically, Methyl-B12, vitamin B6, and 5-Metiltetrahidrofolato are all essential cofactors involved in pathways related to methylation and oxidative stress, and the supplementation with these cofactors showed a significant improvement in ASD symptomatology, especially in a subgroup of ASD children with baseline methylation and antioxidant deficits (132).

Gluten-free diet is often combined with casein-free diet into a single gluten-free and casein-free (GFCF) diet, which has gained popularity in recent years for ASD treatment (133). The rationale for the use of GFCF diet in ASD largely stems from the need to eradicate the effects of some bioactive food-derived opioid peptides, released by the partial digestion of both gluten and casein peptides by intestinal enzymes, such as β-casomorphin-7 (BCM7) among others, with negative consequences on mental health (134). These food-derived opioid peptides can pass through the permeable intestinal membrane, eventually crossing the blood-brain barrier and binding to opioid receptors in the brain, acting as neuromodulators and affecting neurotransmission. Emerging evidence indicates that opioid receptors may indeed be involved in regulating aspects of social behavior, and contribute to the pathogenesis of ASD (134).

In recent years, the literature has reported that besides the human genome, the gut microbiome can also largely influence dietary protein digestion by the expression of peptidases and, thereby, contribute to the change in the host response to the peptides affecting host physiology (135). Indeed, different bacterial strains can degrade food-derived peptides and could consequently be useful to prevent diseases triggered by these peptides. In this regard, bacterial strains commonly found in the infant intestine (e.g., strains of *Bifidobacterium*) have shown particularly high dipeptidyl peptidase IV activity, the primary degrading enzyme of BCM7, suggesting they may limit BCM7 activity during early development. Taking into account the reported evidence and despite the need for further studies, gut microbiota might be considered to positively impact peptidases levels in the context of neurodevelopment and, thus, on ASD. Selected strains of *B. longum subsp. Infantis*, and *B. bifidum*, among others with high degradative capabilities, might be potential probiotic microorganisms able to abolish food-derived opioid peptides and, thereby, contribute to host health (135). Due to the fact that gut microbiota can also impact gluten and casein digestion, it would be interesting to investigate the gut microbiota profile as a potential biomarker for the efficacy of GFCF dietary intervention.

There is still a lack of conclusive evidence for the efficacy of the GFCF diet for ASD treatment, although some positive results have been reported in systematic reviews evaluating the effect of GFCF diet by interventional studies (132, 136, 137). Importantly, the results have described an important time dependency for the detection of beneficial GFCF dietary effects or noticeable improvements. This is probably because gut dysbiosis, inflammation, and perturbations in intestinal motility and permeability encountered in many patients with ASD need a substantial time to normalize after prolonged dysfunction (138). In addition, a high inter-individual variation in response to GFCF diet has been observed (136), and thus studies are warranted to accurately identify biomarkers for patient populations that can benefit from a GFCF dietary intervention.

The Ketogenic diet (KD) is the dietary intervention with the most proven therapeutic effect in Nutritional Psychiatry, especially for the treatment of refractory pediatric epilepsy and with a high therapeutic potential for many mental health and metabolic disorders. The KD has recently been proposed as a promising metabolism-based dietary intervention for the prevention and treatment of ASD. In this respect, a recent systematic review of the literature examining the interplay between KD and ASDs revealed encouraging findings, particularly from animal studies, which support the potential to improve core behavioral symptoms of ASD by using KD or analogous metabolic strategies (139). In fact, various preclinical studies using different animal models have strongly supported the beneficial effect of KD to ameliorate behavioral symptoms associated with ASD (140, 141). In addition, it is important to note that although further studies are warranted to investigate the role of gut microbiota as a potential underlying modulator of the effects of KD on ASD, there are reports of the impact of KD in remodeling the gut microbiome of the BTBR mouse model of ASD (142). Human studies into the potential therapeutic effects of KD in ASDs have also provided promising results (143) although additional studies are warranted to truly understand the short and long-term effects of KD in individuals with ASD, and to investigate the underlying mechanisms.

## Gut Microbiota, Diet, Anxiety, Social Phobia, and Depression

Extensive scientific evidence advocates that unhealthy dietary patterns may increase the risk of developing depression or anxiety, while a healthy dietary pattern and some specific dietary components may do the opposite. This would suggest that dietary interventions could help to prevent, or be an alternative or adjunct therapy for depression and anxiety. However, the relationship between diet and depression and anxiety is complex and further studies are essential to understand the nature of this relationship, especially in individuals vulnerable to these conditions (21). Regarding the effects of specific gut microbiota-related dietary components in anxiety and depression, experimental studies and clinical trials in humans suggest that probiotics and prebiotics might provide anxiolytic effects.

Preclinical studies have revealed potential gut-brain pathways by which gut microbiota exerts beneficial effects on these conditions. For instance, Bravo *et al*. demonstrated that the administration of the lactic acid bacteria *Lactobacillus rhamnosus* in mouse models modulated GABA receptor expression and induced anxiolytic behavioral effects via a vagus nerve-dependent pathway (144). Similarly, Bercik et al. also showed that probiotic intake reduced gut inflammation-induced anxiety and the association of these anxiolytic effects with changes in BDNF, and dependent on the vagus nerve (145). Additionally, in a clinical placebo-controlled trial Tillisch et al. demonstrated that the consumption of a fermented milk product containing a combination of probiotics during a 4-weeks period can modulate brain activity, acting on brain processes of negative social stimuli (146). Additional clinical trials have also demonstrated anxiolytic effects of specific probiotics and prebiotics in patients with chronic fatigue syndrome (147), irritable bowel syndrome (148) and in healthy volunteers from the general population (149).

Regarding the role of diet in preventing the onset of social anxiety disorder or social phobia, a study in 710 young adult students (150) suggested that fermented foods, which typically contain probiotics, may offer some protection against the development of social anxiety disorder in subjects with greater vulnerability to the condition. Although additional research would be necessary to determine the direction of causality, the results advocate that consumption of fermented foods may serve as a low-risk intervention for reducing social anxiety.

## Gut Microbiota, Diet and Schizophrenia

As proposed by the neurodevelopmental hypothesis of schizophrenia (SZ), the abnormal trajectory of brain development linked to this disorder is established during gestation and early life, long before the onset of the clinical symptoms of disease, which often occur in early adult life. SZ is a chronic mental illness characterized by positive symptoms (delusions, hallucinations and disorganized thoughts and speech), negative symptoms (apathy, anhedonia, loss of motivation and lack of social interest) and cognitive alterations (impairments in attention, working memory, or executive function). The disease typically progresses gradually, with early warning signs (prodromal stage) developing before the first severe episode (psychosis) when positive symptoms started to be experienced. Interestingly, the shortened life expectancy observed in patients with SZ has been attributed to many other medical disorders often comorbid to the disease, such as cardiovascular diseases and diabetes.

It is well-established that different early life environmental factors, many of which are linked to gut microbiota shifts, might trigger the onset of this psychotic disorder in genetically susceptible individuals. Indeed, preclinical studies have shown that maternal stress and maternal immune activation (MIA) during pregnancy result in SZ-like behavior in offspring (151). In addition, immune dysregulation and infections have been suggested as crucial risk factors for SZ development (152). Dopamine dysregulation has been traditionally considered as a main etiological factor of SZ and it has been used as a target to develop drugs for managing the condition. However, the use of antipsychotic drugs is often associated with important side effects and, therefore, alternative treatments for SZ are clearly needed. Increasing evidence suggests that gut microbiota may be involved in the development of SZ through immune and inflammatory mechanisms (153). In addition, dopamine can be produced by microbes, and this fact together with the increased gastrointestinal inflammation associated to the disease has strongly suggested the potential role played by gut microbiota in modulating the risk of developing SZ and its prognosis (153).

Recently, a fecal microbiota transplant study showed that the SZ gut microbiome itself can alter the neurochemistry and neurologic function of GF mice in ways that may be relevant to SZ pathology (154). Furthermore, several studies have investigated the microbiome composition in SZ patients compared to healthy control and they identified some significant potential microbiota biomarkers of the disease (155). In addition, it has been suggested that the gut dysbiosis described in SZ patients may affect gut integrity, with increased susceptibility to infection and inflammation and thus contribute to SZ development and its severity. Consistent with this, the abundance of specific bacterial genera, such as *Succinivibrio* and *Corynebacterium*, has been associated with severe symptoms of SZ (156). However, the studies conducted in humans have all been cross-sectional and a causal relationship could not be inferred. Notwithstanding, the potential role played by the gut microbiome in the etiology of SZ strongly motivates the development of microbiome-targeted interventions for SZ prevention and/or treatment. In this respect, there is still inconclusive evidence for the efficacy of pro/prebiotic supplementation in the prevention or treatment of SZ and further research and more clinical trials are needed to test the validity of modulating the gut microbiome to improve the clinical management of SZ.

It is well-recognized that diet plays a key role in controlling inflammation, and research has described the anti- or pro-inflammatory potential of specific foods, dietary components and dietary patterns to be of particular importance (157). In this regard, studies strongly suggest that chronic inflammation, driven partially by dietary factors, might impact on SZ development and aggravate the course of the disease (30). In fact, the dietary inflammatory index (DII), a standardized score related to the inflammatory potential of diet, was reported to be significantly higher in SZ patients in a recent population-scale study (30). Particularly, the intake of total energy, carbohydrates, total fat, saturated fat, and sugar, known as pro-inflammatory components, were found significantly higher in SZ subjects compared to controls (158).

In general, unhealthy dietary intake patterns and increased prevalence of immune and metabolic dysfunction have been observed in subjects with SZ (159). Regarding lifestyle and in the setting of SZ, research has described the relationship between diet quality and other lifestyle factors (physical activity, smoking, and sleep quality). For instance, a correlation between poorer diet quality and smoking in SZ patients has been reported (28). In addition, a pro-inflammatory diet and a poor quality of life could aggravate neuroinflammation and the disease, thereby encouraging the detrimental role of unhealthy foods and poor life-style. By contrast, although clinical trials have not yet evaluated the beneficial impact of the Mediterranean diet or other healthy life-style factors for SZ, some researchers hypothesize that they could improve metabolic and other disease outcomes related to premature mortality in SZ (159).

Some nutritional deficiencies have been proposed to play a role in the etiopathogenic mechanisms of SZ, and particular dietary interventions have been proposed to reverse the SZ-associated neurobiological changes established in the early phases, at least for certain cohorts of SZ patients (160). Regarding nutritional deficiencies, evidence has shown that PUFAs deficits are associated with both negative and positive symptoms of SZ. Additionally, several studies also showed blood levels of PUFAs, particularly DHA, negatively correlated with the severity of SZ symptoms. Nervonic acid (NA) is a monounsaturated omega-9 fatty acid with an important role in myelin biosynthesis, and low NA levels in the red blood cell membrane have emerged as an early risk factor for the development of psychosis in adolescents at high clinical risk (161). Furthermore, omega-3 fatty acid supplementation has been shown to reduce psychotic conversion rates and to improve both positive and negative symptoms as well as global functions in high-risk adolescents (162, 163). Indeed, several randomized double-blind placebo-controlled trials have reported that during the prenatal period, childhood and adolescence, the administration of eicosapentaenoic acid (EPA) and docosahexaenoic acid (DHA) reduce the levels of pro-inflammatory cytokines and ameliorate positive and negative SZ symptoms. However, in patients with chronic psychotic disease, no differences between treatment and placebo groups were observed for psychotic illness improvement, suggesting that omega-3 supplementation would be more effective when prescribed in early life (163).

Regarding micronutrients, several studies have also pointed out particular vitamin deficiencies as risk factors for SZ development. Specifically, folic acid (vitamin B9), piroxidine (vitamin B6), and cobalamine (vitamin B12) deficiencies have been reported common in SZ patients. Total plasma homocysteine (Hcy) level is considered as a sensitive measure of these deficiencies and elevated Hcy levels have been widely associated with the etiology of numerous health impairments, especially cardiovascular diseases and several mental health disorders such as Alzheimer's disease and SZ (164) and some studies have reported a positive correlation between Hcy levels and SZ illness severity (165).

In this regard, several randomized double-blind placebo-controlled trials have shown the effectiveness of homocysteine-reducing strategies, with folic acid and vitamin B supplementation improving both positive and negative symptoms, mostly related to attention/vigilance, and especially in chronic SZ patients with hyperhomocysteinemia (166).

Additionally, low serum levels of vitamin D have also been observed in SZ patients having a negative correlation with psychosis severity (167). Likewise, a randomized, placebo-controlled trial reported a significant improvement in SZ cognitive impairments after 8 weeks of vitamin D supplementation (168). Furthermore, a low dietary intake of vitamin C, which has important anti-oxidative properties, has also been associated with an increased risk of SZ, while vitamin C has been linked to the significant improvement of several SZ-related psychiatric scores (169, 170). Taking into account all the aforementioned evidence, diet should be considered as one of the most crucial factors for the clinical management of SZ patients (160).

Other studies highlight the association between SZ and celiac disease (171) and there have been several small controlled studies in which a GFD showed promising results because approximately 10% of patients ameliorated SZ symptoms. However, large-scale epidemiological studies and clinical trials are needed to investigate the extent and the underlying mechanisms of this gluten-SZ association, as well as to identify biomarkers to predict the clinical response of SZ patients to a GFD (172). Interestingly, it has been reported that patients with SZ tend to eat more carbohydrates immediately before a psychotic episode. This fact points to carbohydrate intake as a potential risk factor for clinical disease progression, and a recent study reported a low-carbohydrate KD as a therapeutic approach to managing longstanding SZ symptoms (173). Subsequently, several case studies have demonstrated that the KD induced significant improvement in psychiatric symptoms, as well as in metabolic dysfunctions observed among patients with SZ (173). In particular, KD has been described to modulate the ratio of key neurotransmitters involved in brain functioning, especially γ-aminobutyric acid (GABA) and glutamate (174). Consistent with this, the modulation of the GABA/glutamate ratio could be a powerful therapeutic mechanism by which KD might act in SZ, since these neurotransmitters may be involved in regulating the altered dopamine levels observed among SZ patients.

## Gut Microbiota, Diet and Epilepsy

There is strong scientific evidence for the role of gut microbiota in seizure susceptibility (175). Firstly, several GF mice studies as well as studies investigating antibiotic-treated mice and humans indicated the specific role of gut microbiota in synaptic changes of key brain areas involved in epileptogenesis. Indeed, Braakman et al. described how five out of six cases of drug-resistant epilepsy became seizure-free during the course of antibiotic treatment (176). Additionally, a prospective study in the pediatric population reported a relationship between rotavirus infections and neonatal seizures. This study also described a significantly close association between probiotic administration after birth and reduction of rotavirus-associated neonatal seizures (177). Furthermore, a recent study also described how gut microbiota transfer by fecal microbiota transplantation (FMT) in rats was able to modulate the risk of seizure, supporting gut microbiota causality (178). This experimental finding was clinically supported by a case study where a patient with Crohn's disease, with a 17-year-long history of seizures, became seizure-free for at least 20 months, despite discontinuing antiepileptic drug treatment with sodium valproate, after a FMT (179). Additionally, several studies have shown patients suffering epilepsy have an imbalance in gut microbiota (dysbiosis), with a greater abundance of potentially pathogenic bacteria such as those belonging to *proteobacteria* (180).

Regarding dietary interventions with a proven efficacy for epilepsy, KD can be considered the most effective diet-based alternative therapy for children and adults with refractory epilepsy. This condition affects more than one-third of epileptic individuals and is defined by a failure to reach seizure control with the currently available anti-epileptic drugs. Additionally, the positive impact of KD has been demonstrated in children and adolescents with refractory epilepsy in terms of mood, as well as social behavior and cognitive functioning, with a beneficial effect on sustained attention and alertness. In a clinical setting, more than half of the patients following a KD respond favorably to this diet, with at least 50% seizure reduction, and different studies have already pointed to some potential biomarkers to predict clinical response to KD in epilepsy (181, 182).

Additionally, several studies in infants and animals with epilepsy have reported the impact of KD on gut microbiota composition (182–184). In this regard, most of the studies concluded that KD triggers important changes in gut microbiota, with a loss of microbial diversity but a positive balance between potentially beneficial genera (such as *Bacteroides* and *Bifidobacterium*) and potentially harmful bacteria (*proteobacteria*). By contrast, in a recent study published by Lindefeld et al., the authors draw attention to the potential negative effects of KD on gut microbiota, describing a decrease in *Bifidobacteria* and an increase in *E. coli* (184).

Some studies have evaluated the beneficial effects that KD-induced modulation of gut microbiota might play in epilepsy. Concerning this issue, a recent study in two mouse models for refractory epilepsy revealed the novel and crucial role for gut microbiota in mediating the anti-seizure effects of the KD (174). This study reported that both GF mice and mice treated with antibiotics were resistant to KD-mediated seizure protection compared with the anti-seizure effects of KD observed in conventionally colonized mice. Furthermore, the authors identified specific members of the intestinal microbiota with essential roles in mediating the KD-anticonvulsant effects. Specifically, authors demonstrated that KD intervention modified gut microbiota composition by significantly increasing the bacterial species *Akkermansia muciniphila* and *Parabacteroides merdae* and, most remarkably, they demonstrated that the gnotobiotic colonization of GF mice with these two bacteria conferred similar protection against epileptic seizures as KD (174). The authors also investigated the underlying mechanisms of the effects of combined administration of *Akkermansia muciniphila* and *Parabacteroides merdae* and found an increase in the GABA/glutamate ratio in the hippocampus, which could explain the changes observed in behavior. These findings have not been confirmed in humans yet, but if established, this microbiota-based intervention strategy (*Akkermansia muciniphila* and *Parabacteroides merdae* co-supplementation) could be recommended to replace KD for refractory epilepsy treatment. Although KD is highly valued to treat epilepsy and has therapeutic potential for the prevention and treatment of other mental health and metabolic disorders (185); its widespread implementation remains limited due to the potential long-term side effects (143). We have yet to clarify the precise mechanisms by which KD exerts its beneficial neuroprotective and metabolic effects in certain disorders, and studies are needed to identify biomarkers for clinical responses to KD, as well as those related to KD side-effects. The mechanisms by which KD appears to exert its anti-seizure effects seem to differ from those by which antiepileptic pharmacological treatments act, suggesting that KD might also be considered as an adjuvant treatment to improve disease management (175).

Regarding the use of probiotics, a study in an animal model of epilepsy known as the Pentylenetetrazol-kindling model found that the administration of other combinations of different probiotic strains (*Lactobacillus rhamnosus, Lactobacillus reuteri*, and *Bifidobacterium infantis*) in PTZ-kindled rats, reduced seizure severity and epileptic activity when compared to seizures in controls (186). Interestingly, in this study the anti-epileptic effects of probiotic administration were accompanied by an increase in GABA levels in the brain, as well as a decrease in brain oxidative stress. This was the first preclinical report showing a positive effect of probiotic bacteria on seizure-induced neurological alterations such as learning difficulties and memory deficits.

In another recent study in humans, a probiotic treatment was shown to reduce seizure frequency by 50% in 29% of patients with drug-resistant epilepsy (187). Furthermore, different studies have reported that the gut microbiome of patients with drug-resistant epilepsy differ from that of patients with drug-sensitive epilepsy. Specifically, an increase in alpha diversity has been described, with a greater relative abundance of rare bacteria, mainly belonging to the phylum Firmicutes, in drug-resistant patients compared to drug-sensitive patients and healthy controls (180). Furthermore, an inverse correlation was reported between the relative abundance of *Bifidobacteria a*nd *Lactobacilli* and the number of seizures per year in both patient groups.

It is noteworthy that the intestinal microbiota is increasingly recognized as an important factor in biotransformation of xenobiotics, including medications (188, 189). Regarding the role of the gut microbiome in the response to antiepileptic drug therapy, several studies have reported that Zonisamide, a common antiepileptic drug, is metabolized to 2-sulfamoylacetylphenol by the intestinal microbiota (190), supporting the role of gut microbiota in modulating anti-epileptic drug metabolism and, thus, in the clinical response to anti-epileptic drugs. In turn, treatment with anti-epileptic drugs may also impact on gut microbiota composition. More specific studies are needed to investigate the true significance of the interaction between anti-epileptic drugs and the intestinal microbiome in epilepsy treatment.

## Towards “Precision Nutrition” in Psychiatry and Neuropediatrics: Where Are We Now and Where Should We Go?

**Figure 3:**
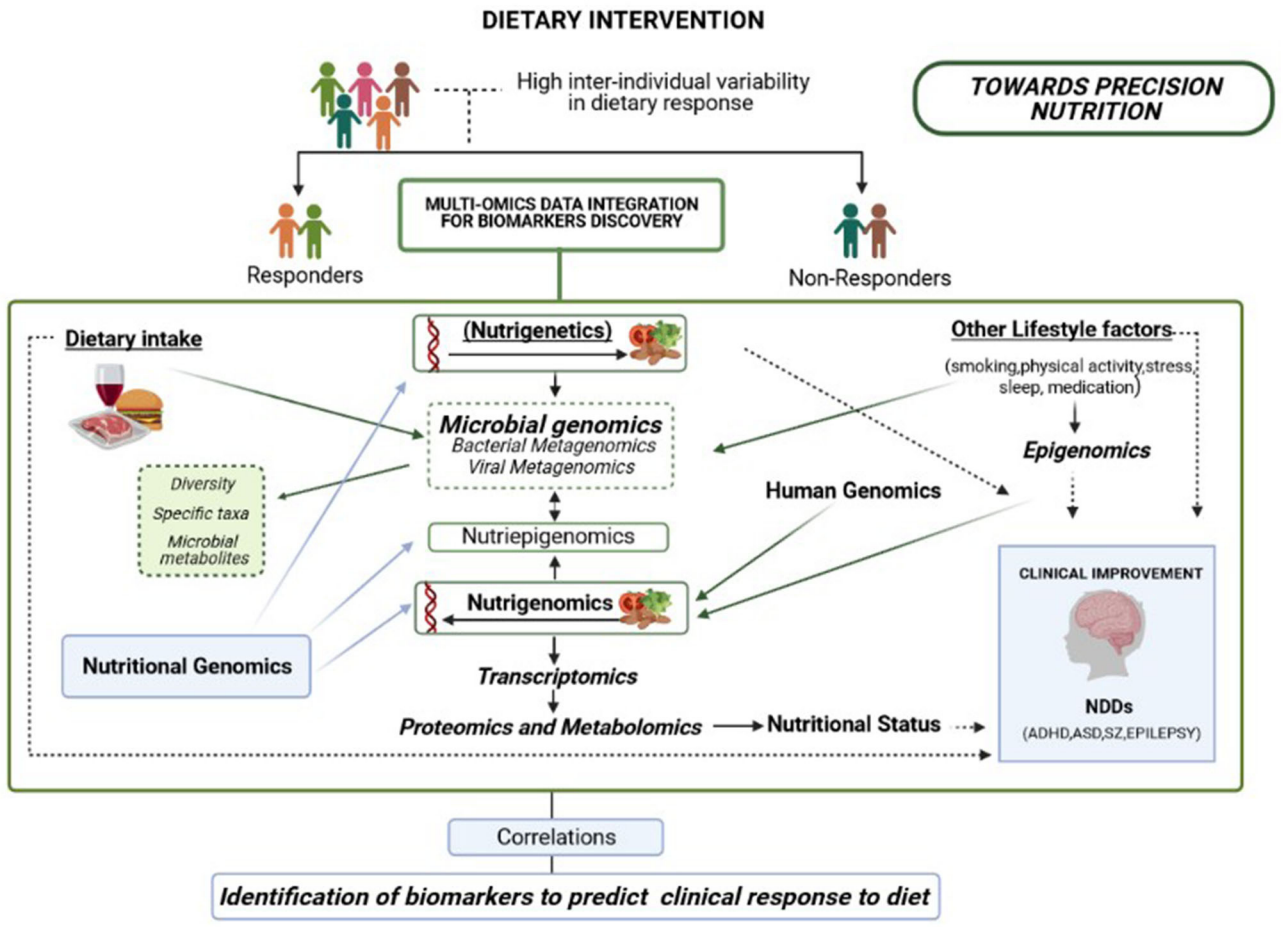
Overview of a multi-omics integration analysis to reveal biomarkers able to predict clinical response to diet, including genomics, metagenomics, epigenomics, transcriptomics, proteomics, and metabolomics. An integrated approach is necessary to improve the effectiveness of dietary interventions aiming to promote healthy brain development according to multiple individual features, including baseline microbiome signatures.

It is important to highlight that even though there are food intake guidelines and general dietary recommendations for the population as a whole, a high inter-individual variation has been documented in clinical response to diet (191). The concept of “Precision Nutrition” arose when the scientific community agreed that the observed inter-individual variability in response to diet was not exclusively based on genetics (nutrigenetics), but rather on the interaction between the host's genetic make-up and the environment (192, 193). Regarding Nutritional Psychiatry, scientific evidence strongly supports that subject stratification based on multiple individuals' specific variables, including gut microbiota profile modulated by multiple factors such as diet and other lifestyle factors (physical activity, smoking, medication and other features), would increase the effectiveness of particular dietary interventions for improving mental health (Figure 3).

To highlight the relevance of the gut microbiota profile for precision nutrition, in the context of cardiovascular diseases (CVDs) often associated to mental health disorders, researchers few years ago reported the role of the gut microbiota in modulating the relationship between red meat consumption and atherosclerosis and other cardiovascular disorders. In particular, several studies in mice and humans have reported increased fasting plasma levels of trimethylamine (TMA), a compound produced by the gut microbiota metabolism, and its proatherogenic metabolite trimethylamine-N-oxide (TMAO), concomitant with an increased risk of atherosclerosis, after oral intake of L-carnitine and phosphatidylcholine depending on the gut microbiota profile (194). Additionally, an algorithm that integrates gut microbiota profile and other parameters, such as blood, dietary habits, anthropometrics and physical activity, was devised to accurately predict personalized postprandial glycemic response to real-life meals (195). Thus, tailored dietary strategies may successfully modify elevated postprandial blood glucose and its metabolic and mental health consequences.

Of note, relevant also for the precision nutrition field, is the fact that the interaction between diet and host genetic background likewise modulate gut microbiota composition. In this regard, a recent study including three independent Dutch population cohorts found a gene-diet interaction between a functional variant at the lactase locus and the intake of dairy products modulating *Bifidobacterium* abundance (196), an important aspect to be considered in Precision Nutrition. Accordingly, in the context of Nutritional Psychiatry, several studies have been conducted aiming to design tailored dietary and gut microbiome-based interventions to promote the development and maintenance of a healthy brain. Remarkably, regarding different responses to the effect of the omega-3 PUFA supplementation on mental health, a direct link was reported between lower endogenous PUFA levels associated with the inflammatory status and an increased risk of developing interferon-alpha-induced depression (197). In addition, pre-treatment with EPA was demonstrated to reduce the onset of depression induced by this inflammatory cytokine (198). In this regard, Rapaport et al. found antidepressant effects of EPA administration only in patients with high baseline inflammation, indicating that the effective supplementation of EPA might depend largely on the pathogenesis and underlying mechanisms of depression (199).

Consistent with this framework, another recent study found that youths with the lowest levels of baseline endogenous EPA show the largest improvement in cognitive function following EPA administration (29). Also, two clinically distinct endophenotypes in SZ patients determined by PUFA levels have been described and SZ patients with low PUFA levels have more negative symptoms than those with high levels, and they show a better clinical respond to omega-3 intervention (200). In addition, a study reported that the attention improvements in ADHD children with EPA supplementation appear to occur more frequently in children with comorbid ODD (201), children who respond poorly to medication, and children with comorbid learning difficulties (202). This would suggest that ADHD-specific symptoms may respond differently to a dietary intervention based on the presence of comorbid disorders. Moreover, an ADHD subgroup, particularly with high inflammation or low baseline levels of LC omega-3 PUFAs, may profit more from omega-3 PUFA supplementation (29). Some studies have already indicated that the inter-individual variability in omega-3 PUFA clinical response seems to be largely explained by genetic variants and epigenetic modifications located within human genes involved in PUFA metabolism and associated with the risk of ADHD and ASD, such as *FADS1, FADS2, ELOVL5*, and *ELOVL2* (203, 204).

Genetic and epigenetic variations in these genes related to the long chain -omega-3 PUFA endogenous synthetic pathway has been directly associated to serum and tissue levels of these fatty acids (205, 206). The epigenetic modulation of gene expression is related to many environmental factors, therefore the environment may shape the individual's ability to metabolize and accumulate omega-3 PUFAs in tissues. Interestingly, the ability of gut microbiota to produce PUFA-derived bacterial metabolites was also reported some years ago, while recent studies have highlighted the important role of gut microbiota in the metabolism of PUFAs (207). Particularly, Djuric *et al*. investigated whether the individual microbiota profile may influence the efficacy of omega-3 PUFA supplementation in reducing colonic prostaglandin E2, and they found a subgroup of individuals showing high abundance of *Prevotella* who were resistant to omega-3 PUFA anti-inflammatory effects (208). This observation strongly suggests that the individual gut microbiota profile may be an additional factor able to influence the efficacy of omega-3 PUFA supplementation. Moreover, variable intake of non-lipidic dietary factors has also demonstrated to affect PUFAs metabolism. The metabolic antagonism of omega-3 PUFAs by high levels of linoleic acid (LA) is well-known, as is the correlation between low levels of linolenic acid (LNA) and the scarce production of EPA and DHA omega-3 PUFAs. On the other hand, high levels of EPA and DHA lead to feedback repression of omega-3 PUFA synthesis (209). Thus, based on the current evidence, it is fundamental to highlight the need for future studies to consider baseline PUFA status together with other features, such as age, sex, and genotype among others, which may modulate the effects of omega-3 PUFAs on behavior, mood and cognition. Taking into account all the above, the final goal should be to identify specific factors/biomarkers or rather the network of factors that could accurately predict those subjects that might truly benefit from sustained long-chain omega-3 supplementation in clinical practice.

Regarding the role of gut microbiota on modulating the effectiveness of KD, Zhang *et al*. described how gut microbiota could be considered as a biomarker for KD efficacy in epilepsy. Specifically, authors reported some microbial taxa such as *Clostridiales, Ruminococcaceae, Rikenellaceae, Lachnospiraceae*, and *Alistipes* related to a poor KD clinical efficacy. Additionally, some genetic variants have also been associated with the neurological outcomes and the degree of weight loss in KD intervention studies (210) and studies have cited baseline ketones levels in blood as potential biomarkers for the KD response (181). It is thus very clear that both genetic and dynamic markers of KD response may help to identify individuals most likely to benefit from KD and to pinpoint individuals at higher risk of adverse KD-related health outcomes (biomarkers of contraindication for KD intervention). The underlying mechanisms of gene–microbiota-diet interactions involved in KD response are still unknown but they might involve differences in KD-mediated gene expression changes in the brain. In this regard, KD-induced improvements in SZ symptoms are reported to be accompanied by reduced levels of TNF-α in plasma and IL-1β in the brain (211), which would suggest the more favorable effects of KD in SZ patients with comorbid inflammation. In conclusion, future multi-center and long-term studies are warranted to accurately identify all KD response biomarkers, including those related to adverse effects, for all the mental disorders that may benefit therapeutically from KD.

Regarding the effectiveness of homocysteine-reducing strategies for the treatment of SZ, different factors has been largely linked to the efficiency of Hcy catabolism, such as the availability of folic acid, vitamin B12 and vitamin B6, and to the modulation of the response to the dietary intervention. In addition, the presence of different common polymorphisms (C677T and A1298C) located in the methylenetetrahydrofolate reductase (*MTHFR*) gene, which modulates MTHFR enzyme activity, and other factors such as age, gender, smoking and certain medications have been associated with Hcy levels (212). In consistency with this, a recent study reported that female SZ patients with hyperhomocysteinemia and affective psychosis were associated with greater improvements in attention/vigilance following B-vitamin supplementation (213). Thus, further studies are needed to identify all the individual features that could, when combined, interact with folic acid and B-vitamin treatment, determining specifically intervention outcomes.

Regarding gluten-free and casein-free (GFCF) regime as therapeutic approach for ASD, a recent systematic review of four randomized controlled trials on GFCF diets found a 50% efficacy rate of treatment, and reported that the GFCF diet may provide more substantial clinical benefits in individuals with low GSH levels and in those with preexisting GI symptoms (136). Research into gut microbiota shows its strong involvement in the digestion of both gluten and casein (135) and this indicates the importance of analyzing the gut microbiota profile as a potential biomarker for the efficacy of GFCF dietary interventions. In this respect, further studies are warranted to identify all biomarkers, including gut microbiota, which may help to accurately predict patients able to benefit from a GFCF dietary intervention. Regarding the therapeutic effects of diet in ADHD, recent double-blind research investigating the effects of a few-foods diet (FFD) demonstrated a significant decrease in ADHD symptoms in many patients adhering to this particular dietary approach. Indeed, in the most recent randomized controlled trial, 64% of children with ADHD responded favorably to the FFD, with its use being strongly recommended in cases of ADHD comorbid food allergy symptoms (27). Currently, ongoing research is focusing on the identification of baseline molecular signatures or biomarkers that could help to accurately predict whether FFD participants will belong to the FFD-responder or non-responder group. Likewise, ongoing research also aims to provide a better understanding of the mechanism(s) underlying the FFD response and to study the role of gut microbiota changes in modulating the effects of this dietary approach. The research underway is based on an unbiased approach, integrating multiomics data of microbiota-gut-brain axis parameters, and including profiling of the microbiome, transcriptome, metabolome, methylome and proteome. Results could enable researchers to accurately identify molecules that might (co)determine FFD effects based on the individual's microbiota-gut-brain (MGB) axis configuration.

## Conclusions, Knowledge Gaps, and Future Perspectives

Currently, preclinical and cross-sectional studies have strongly established that the gut microbiota influences cognition, mood, and behavior. In this regard, research highlights that early life perturbations of gut microbiota function can profoundly impact neurodevelopment, with potentially crucial long-term consequences on health. However, there is still a critical need for population-based prospective cohort studies to provide a comprehensive understanding of the early temporal variability in the host-microbe dynamic interactions, which determine the shift from mental health to mental disorder, and vice versa. In this regard, gut microbiota profiles and determinants of those profiles linked to the onset of NDDs should be extensively investigated, and further studies are essential to design gut microbiome-targeted intervention strategies to promote the development and maintenance of a healthy brain. Thus, to develop more effective microbiome-based interventions and innovative dietary supplements targeting NDDs, we require a greater understanding of the beneficial gut microbiota members for mental health. These would include bacteria as well as bacteriophages, which are active against particular bacteria, and the underlying mechanisms by which they act, including bioactive molecules (postbiotics). In the near future, such potential novel intervention strategies could pave the way toward preventing the development of NDDs in subjects with risk indicators, and provide new alternatives to improve the clinical management of subjects diagnosed with NDD.

Diet is also considered as an amendable factor that can help to protect against the onset and manifestation of mental health disorders by controlling the gut-brain axis. To date, studies have reported a prominent inter-individual variation in the clinical response of NDDs to beneficial dietary interventions, and consequently a major challenge currently faced by nutritional interventions is to identify biomarkers that can accurately predict the efficacy of the response.

The gut microbiota may be a key mediator of dietary effects on mental health, although diet is also known to be involved in brain functioning through other microbiota-independent mechanisms. In this respect, exhaustive studies are needed to decipher the precise mechanisms by which certain dietary interventions exert their effects, with the main goal of designing safer and easy-to-implement targeted strategies, based on those underlying mechanisms. Finally, it is also important to note that although diet is a major component of an individual's lifestyle, data on other lifestyle factors and the Human Genome provide fundamental information that should also be taken into account when studying the determinants of mental health, besides the bases of dietary effects on mental health. In fact, scientific evidence has indicated that the effects of diet on the host may be modulated by variations in the human genome as well as by other lifestyle factors that may, likewise, influence gut microbiota and human genome expression. Therefore, in the current -omics era, further studies based on multi-omics data integration are greatly needed in the field of Nutritional Psychiatry in order to identify baseline molecular signatures and/ lifestyle determinants (diet and other lifestyle factors) that may (co)determine or predict the specific effects of dietary interventions. The results provided by these studies will likely lead to refined future dietary strategies to prevent or improve the treatment of NDDs, and, in this way, help Psychiatry progress towards tailored nutrition and personalized medicine.

## Author Contributions

MC and AL wrote the first draft of the manuscript. JP, PC-F, and MC have contributed substantially to the writing and revising of the manuscript. MC designed the aim of the editorial. All authors have made substantial intellectual contributions, took responsibility to the manuscript, and give final approval of the version to be submitted.

## Conflict of Interest

The authors declare that the research was conducted in the absence of any commercial or financial relationships that could be construed as a potential conflict of interest.
